# Patient perspectives on barriers to care and the perceived value of a direct-to-patient virtual learning program for chronic intestinal failure

**DOI:** 10.1016/j.intf.2026.100390

**Published:** 2026-08-07

**Authors:** Elisa Fisher, Marion Winkler, Malvika Nair, Princess B. Ballog, Marjorie Nisenholtz, Constantin T. Yiannoutsos, Rocco Friebel, Kishore R. Iyer

**Affiliations:** a Center for Evaluation and Applied Research, New York Academy of Medicine, New York, NY, United States; b Rhode Island Hospital, Brown University Health, Providence, RI, United States; c Recanati/Miller Transplantation Institute, Mount Sinai Hospital, New York, NY, United States; d Department of Epidemiology and Biostatistics, City University of New York, New York, NY, United States; e Department of Health Policy, London School of Economics and Political Science, England, London, UK; f Intestinal Rehab & Transplant Program, Mount Sinai Medical Center, Icahn School of Medicine at Mount Sinai, New York, NY, United States

**Keywords:** Chronic intestinal failure, Patient engagement, Access to care, Disease management, Project ECHO, Qualitative research

## Abstract

**Background:**

There is a shortage of clinical expertise in chronic intestinal failure (CIF). We aimed to explore patient perspectives on the need for an evidence-based virtual tele-learning program to train patients in best practice CIF care using the ECHO™ Model. Findings were used to inform development of the Patient Intestinal Failure-ECHO (PIF-ECHO) Project.

Material and Methods: Using a qualitative research design, this study included interviews with six CIF patient advocates and two virtual focus groups with nine individuals living with CIF who expressed interest in PIF-ECHO. Data were coded and analyzed using iterative thematic analysis to generate themes focused on perceived need for and value of PIF-ECHO.

**Results:**

Four main themes were identified: 1) lack of provider access and support for patients living with CIF, 2) high levels of patient responsibility for disease management, self-advocacy, and system navigation, 3) limited access to patient-facing information on best practice CIF care, and, 4) severe emotional strain. Perceived value of PIF-ECHO centered on the program’s potential to improve knowledge and access to resources, which participants hypothesized would lead to improved capacity for disease self-management, and ability to advocate for their needs across healthcare settings. Findings informed the development of a PIF-ECHO logic model illustrating connections between program activities and anticipated outcomes.

**Conclusion:**

A patient-facing ECHO program for CIF has the potential to address patient-identified needs related to access to specialist expertise and patient education, ultimately improving CIF patients’ ability to manage their own care and advocate for their needs across systems.

## 1 Introduction

Chronic intestinal failure (CIF) is a rare and devastating condition in which individuals cannot absorb nutrients and water required to meet their needs. Patients with CIF are dependent on home parenteral nutrition (PN) administered intravenously to provide nutrients required to maintain health [1], [2], [3], [4]. PN is administered over many hours each day and requires patients to have an indwelling central venous catheter and be tethered to an infusion pump, which has a significant impact on quality of life [5], [6], [7], [8], [9], [10]. While lifesaving, long-term PN is complicated by severe morbidities, including episodes of dehydration, central line-associated bloodstream infection (CLABSI), and intestinal failure-associated liver disease all leading to increased hospitalizations, healthcare costs, and mortality [11], [12], [13], [14], [15], [16], [17], [18], [19], [20].

There is wide recognition that the complex care of CIF patients is best delivered through multidisciplinary teams operating in intestinal rehabilitation programs (IRP), yet over half of all states in the United States and many rural communities lack specialty centers [12], [21], [22], [23]. The problem of health inequity in CIF is likely multi-factorial as disparities in socioeconomic status and access to care compound challenges associated with disease rarity, chronicity and acuity, high costs of care, and a critical shortage of widely available expertise.

To address the significant challenges stemming from lack of expertise in CIF, the well-established Project ECHO® (Extension for Community Healthcare Outcomes) Model [24], [25], [26], [27], [28], [29], [30], [31], [32], [33] was adapted and launched as the *Learn Intestinal Failure Tele-ECHO Project* (LIFT-ECHO). The goal of the virtual tele-mentoring platform is to disseminate knowledge and best practices to non-specialist clinicians and improve outcomes in CIF [34], [35]. Despite high engagement in LIFT-ECHO, preliminary data suggests that the majority of participants already possess CIF expertise and very few non-specialist clinicians participate. In its current form, the ECHO Model is limited to training professionals and prohibits patient participation.

To address these gaps, the *Patient Intestinal Failure-ECHO Project* (PIF-ECHO)—a novel virtual learning model designed to provide multidisciplinary education and support directly to CIF patients and their family caregivers—was created and is undergoing pilot testing (AHRQ:1RO3HS030321–01). Modeled on the LIFT-ECHO program, the program aims to enable patients to learn best practices in CIF care from expert clinicians and from each other’s lived experiences, ultimately increasing the knowledge and self-efficacy required for managing their condition.

Given the importance of incorporating patient perspectives into the development and implementation of healthcare interventions [36], [37], the aim of this study was to gain a greater understanding of patient perspectives on key challenges common among patients with CIF to inform the design and implementation of PIF-ECHO, as well as on the potential value and anticipated outcomes of the program.

## 2 Methods and materials

This study used qualitative descriptive research [38] to explore patient perspectives on key challenges faced by patients living with CIF, the potential value of the PIF-ECHO program in addressing those challenges, and implementation considerations.

The protocol was led by an evaluation team of two people, including a lead researcher experienced in qualitative research methods and mixed methods utilization-focused program evaluation (EF), as well as a supporting researcher with training in qualitative research methodologies and experience in program evaluation (MN). CIF specialists included a surgeon who directs an intestinal failure and rehabilitation program and serves as the Principal Investigator (PI) leading the development and implementation of the PIF-ECHO program (KI) as well as a clinician with expertise in PN and quality of life (MW). CIF specialists provided support throughout the study, including planning, patient recruitment, and interpretation of findings.

All study participants provided verbal consent for taking part in the study and received modest incentives for their participation. Ethics approval has been obtained from the New York Academy of Medicine Institutional Review Board (IRB) with the letter number.

#011426 dated January 20, 2026, and from the BRANY IRB with letter number STUDY−24–01292 dated November 12, 2025.

### 2.1 Participant recruitment

#### 2.1.1 Interviews

Qualitative interviews were conducted with a purposive sample of six patient advocates who focus on rare disease, CIF, home PN, and caregiving. Interviewees were selected due to their roles as leaders or administrators of national CIF-related advocacy organizations or Facebook support groups; each also had lived experience related to CIF, either personally or as a caregiver. Patient advocates were invited to participate in interviews via an email from the PI leading the PIF-ECHO initiative (KI), who then referred interested participants to the evaluation team.

#### 2.1.2 Focus groups

Two virtual focus groups were conducted with individuals living with CIF. Focus group recruitment was driven by administrators of private CIF-focused Facebook groups posting information about the intervention on their site. Individuals who expressed interest were given additional information on the proposed program; those who agreed to participate were invited to join a planning-oriented focus group via email from the PI leading PIF-ECHO (KI). Contact information for those interested was then shared with the evaluation team, who sent the focus group link to confirmed participants.

### 2.2 Data collection

#### 2.2.1 Interviews

The semi-structured interview guide, developed by the research team with input from CIF-specialists and one patient advocate, included questions exploring access to care, information, and social support among individuals with CIF, as well as perceptions and recommendations related to the proposed PIF-ECHO program. Interviews were conducted and recorded via Microsoft Teams videoconference software. One patient advocate was also part of the research team and had previously met the interviewer; no other participants had a prior relationship with the interviewer. Interviews took place between January and February 2026 and lasted an average of 65 minutes.

#### 2.2.2 Focus groups

The semi-structured focus group topic guide included questions on experience accessing healthcare, perceived need for and access to CIF-related information support, and recommendations related to the proposed PIF-ECHO program. One focus group took place in March 2026 (n = 5) and a second took place in April 2026 (n = 4). Both were hosted using Zoom teleconferencing software and lasted approximately 90 min. Groups were led by a facilitator (EF), primarily responsible for guiding the overall discussion, and a co-facilitator (MN), responsible for notetaking, recording attendance, timekeeping, and addressing any technology-related issues as they arose. Facilitators had no prior relationship with focus group participants.

### 2.3 Data analysis

Recordings of both interviews and focus groups were professionally transcribed, and transcripts were managed and analyzed using Nvivo software (Version 15). Data were analyzed using an iterative thematic analysis approach consistent with Braun and Clarke’s 2006 framework for reflexive thematic analysis and the qualitative descriptive approach described by Colorafi and Evans [38].

More specifically, notes and transcripts were initially reviewed by the evaluation team with topics and sections of text organized according to key topics derived from both the research questions and the data itself (EF, MN). These preliminary themes and the associated excerpts were reviewed by the expert team of CIF specialists (KI, MW) and were used to develop an initial codebook. Initial codes were then applied to a sample of transcripts (n = 3), at which point additional analytic discussions within the evaluation team facilitated clarification and refinement of the coding scheme. Previously coded transcripts were then updated using the refined coding scheme, which was also applied to remaining transcripts. The final codebook included both deductive themes designed to inform program design and implementation as well as inductive themes that emerged during data review and coding processes.

Analysis involved repeated reviews of coded extracts by evaluation team members to identify connections between topics and generate high level, data-supported themes and subthemes, as well as thematic review and discussion with content experts (KI, MW) to ensure credence to the data analysis process, confidence, and credibility. Deeper analysis and interpretation of qualitative data led to the development of a logic model visualizing hypothesized connections between patient priorities, program activities, and anticipated program outcomes. The model, developed in collaboration with the PIF-ECHO program team, translated key themes derived from interviews and focus groups into a framework describing the anticipated pathways through which program activities are expected to lead to participant outcomes. Initial findings and the resulting logic model informed future program implementation and evaluation efforts.

Sample quotations pertaining to each theme and the pathways illustrated in the PIF-ECHO logic model are presented in the Results section. An ellipsis (…) is used to indicate omitted words or phrases, and brackets [] are used to designate words added for clarity.

## 3 Results

### 3.1 Participant demographics

The focus groups included nine adults, all female, white, with a mean age of 47.2 + 14.3 years. Two thirds (n = 6) had a primary diagnosis of short bowel syndrome; the remainder had intestinal dysmotility (n = 2), and/or intestinal fistula, small bowel mucosal disease, Crohn’s disease, or Hirschsprung disease in their history. All participants received home PN with a mean length of time on therapy of 6.35 + 4.36 years. Most participants had either Medicare (n = 4) or private insurance (n = 4). Participants lived throughout the United States, with three in the West, three in the South, two in the Midwest, and one in the Northeast. The majority (n = 7) of participants did not receive CIF care at an IRP. Demographic information for focus group participants is summarized in Table 1.

**Table 1:** Demographics of focus group participants.

	**N**
**Total**	9
**Gender**	
Female	9
**Age range**	
18–34	1
35–54	5
55 +	3
**Race**	
White	9
**Geographic location**	
West	3
South	3
Midwest	2
Northeast	1
Insurance*	
Medicaid	1
Medicare	4
Private	4
Other	1
**Years on PN**	
0–5	4
5–9	3
10 +	2
**Receives care at IRP**	
No	7
Yes	2

IRP=Intestinal Rehabilitation Program.

PN= parenteral nutrition.

* Multiple responses permitted

## 4 Common challenges experienced by patients with CIF

The aim of this study was to gain a greater understanding of patient perspectives on key challenges common among patients with CIF to inform the design and implementation of PIF-ECHO, as well as on the potential value and anticipated outcomes of the program. Four main themes were identified, including 1) lack of provider access and support for patients living with CIF, 2) high levels of patient responsibility for disease management, self-advocacy, and system navigation, 3) limited access to patient-facing information and education on best practice CIF care, and consequently, 4) severe emotional strain experienced by patients with CIF.

### 4.1 Lack of access to and support from healthcare providers

#### 4.1.1 Limited provider expertise and availability

Interview and focus group participants alike consistently and frequently described a severe lack of supportive resources to help them navigate and manage their CIF. Limited expertise among healthcare providers across a wide range of specialties hinders patient access to needed care and their ability to understand and manage their condition. There is widespread recognition that obtaining higher quality care requires access to CIF specialists, however, participants described numerous barriers in accessing the few CIF specialists who exist, including distance, long waitlists, insurance and cost limitations, and provider turnover. As a result, many patients with CIF struggle to find a provider who will accept their case, and many rely on providers who lack the knowledge necessary to treat their condition.

*Even though you’d think [the academic medical center] would be a pretty good place for care, they just weren’t super knowledgeable. I’ve had multiple people there tell me intestinal failure doesn’t exist and it’s not real. (Focus group participant)*

*In terms of specialists who are familiar with short bowel…there is one in [my whole state], and I had to wait a year to see her. And she’s older, and every day I fear she’s going to announce her retirement. And then, I don’t know where that leaves the whole program. (Focus group participant)*.

#### 4.1.2 Patient-provider relationship dynamics

Challenges related to patient-provider relationships were frequently described as barriers to effectively managing and advocating for care. Participants explained that providers are often hesitant to trust patients or are skeptical when they describe symptoms; many described experiences of being ignored, dismissed, or not believed in healthcare settings. Participants also discussed fears and actual experiences of being dropped by providers due to their medical complexity or perceived adversarial behavior. Thus, for those without access to a supportive team of specialists, interactions with healthcare providers were seen as a source of stress rather than a supportive resource.

*There is a group of [providers] who’ll listen [to patients], but the vast majority just don’t even listen. They dismiss us all together as you don’t know what you’re talking about. They just don’t hear you. (Advocate)*

*If your needs are too high, or you have multiple emergencies, or problems between appointments, and you contact the office too often, and the provider feels overwhelmed, a lot of times they do. They’re like, “You need to find somebody else who’s more specialized, or who can manage this.” And so, I tend to not want to bring up problems that I’m having because I don’t want that to happen again. (Focus group participant)*

### 4.2 Patient burden for treatment administration, care coordination, and self-advocacy

#### 4.2.1 Disease and therapy self-management

Participants frequently discussed challenges associated with the high levels of patient responsibility inherent in disease therapy for patients with CIF, noting that patients with CIF often must engage in activities typically limited to medical professionals. They discussed administering and adjusting their own PN, changing their own central line dressings, doing their own lab draws, and compounding their own medications at home, often with very little support from providers. Feelings of self-efficacy vary; those well-connected to CIF specialty care and those with more experience expressed greater confidence in their abilities.

*This cohort of patients [with CIF] is unique compared to other disease diagnoses and therapies because for all the other things, I can rely on my doctors, and they can do all the things that they need to…But for this world, we do 24/7 care most of the time. (Advocate)*

#### 4.2.2 Clinical care advocacy

Due to the complexity of their illness and limited access to CIF specialists, participants explained that patients with CIF are often forced to take greater ownership of coordinating their care across providers and systems compared to patients with more common chronic conditions. Participants described multiple experiences in which they were responsible for tasks handled by providers, such as researching their condition, identifying specialists to be a part of their care team, exploring and suggesting treatment options, and coordinating care across specialists.

*I had a drug-resistant infection, got really sick… I’m the one who had to figure out [what] the next step would be …no one takes ownership of care. It’s only you. I even went to my PCP and said, “I need help. This is getting to be a lot.” And he’s like, “I’m sorry I can't help you more.” (Advocate)*

#### 4.2.3 System-related advocacy

Participants also described experiences in which they had to advocate during interactions with health system administrators, infusion companies, and insurance companies. They shared specific examples of pushing back on health system and infusion company policies that limit access to needed care or resources or that harm their quality of life. They also discussed experiences working to overcome barriers to insurance coverage, noting that their requests for pre-approval or submitted healthcare claims are denied.

*It’s bills being denied and then having to do your own…self-advocacy…going back to the other hospitals, going, “Hey, can you look at these codes to make sure that it is, because when I got pre-approved, it was all covered. And then, now it’s not, because it was billed wrong.” (Focus group participant)*

#### 4.2.4 Lack of patient education and training

According to participants, heightened patient responsibility for understanding their health needs, administering CIF-related treatment (e.g., home PN), and advocating for their own care necessitates greater patient access to highly specialized information compared to other chronic diseases. However, participants reported insufficient access to quality information designed for patients. This leads many to seek information, support, and advice from both informal (e.g., AI, social media/online forums) and formal online resources (e.g., national advocacy organizations), which offer varying degrees of utility and accuracy.

*Where does the information [for CIF patients] come from? You could look on a website, get information, it could all be fake or AI generated or whatever. And that’s not what we’re looking for. We’re looking for real, experienced—real education pieces. (Advocate)*

Some participants specifically suggested a need for more or better training related to administering their own care, given the high-level at-home responsibilities that fall on CIF patients and their caregivers. Experiences of training varied, with some participants feeling well-informed and confident in their ability to manage their own care and others feeling inadequately prepared.

*In adult care, I see a lot more patients being sent home with inadequate training about their central lines, where they’re kind of left to fall back on their infusion providers for training. And that may or may not be adequate. (Advocate)*

#### 4.2.5 Emotional strain

Participants discussed significant emotional strain resulting not only from the chronicity and severity of their disease, but also the responsibility of researching and administering their own care, communicating with and maintaining relationships with providers, navigating the health system, and advocating for their needs. Emotional strain included feelings of stress, anxiety, being overwhelmed, frustration, as well as a sense of desperation for answers, support and empathy.

*To be able to keep advocating and keep speaking up is exhausting. And it’s exhausting physically. It’s exhausting mentally. (Focus group participant)*

### 4.3 Participant perspectives on PIF-ECHO and data-driven PIF-ECHO logic model

As noted previously, all focus group members and interviewees expressed interest in the proposed patient-facing ECHO program prior to participation in this study. Interview and focus group participants consistently suggested that PIF-ECHO has the potential to address aforementioned challenges related to lack of patient education and the gap in patient-facing resources in best practice care, necessitated by systemic barriers to expert care and the high level of patient responsibility for disease management, advocacy, and system navigation. They emphasized that the value of the PIF-ECHO program was centered in its ability to increase access to trusted, quality information on best practice CIF care, suggesting two key pathways for impact on health: 1) improved patient capacity for disease self-management, and 2) increased patient empowerment related to communicating about and advocating for their needs across healthcare settings.

*I mean, we’re already talking about how we’re kind of in charge of so much of our own care anyway and have to make so many decisions… I feel like the more knowledge I have, the more empowered I am to take care of myself better. – Focus group participant*

*Where when you do an ECHO with a physician, you reach one physician. When you do an ECHO with one patient, you’re training a patient who will be able to advocate for themselves in the emergency room, in the GI’s office, in the primary care office. You’re providing that patient with the knowledge that they need to be able to seek care in every healthcare setting that they will be in. And because CIF affects them across all of those settings, I think that it will provide them with more consistent care. – Advocate*

### 4.4 PIF-ECHO logic model

**Fig. 1:**
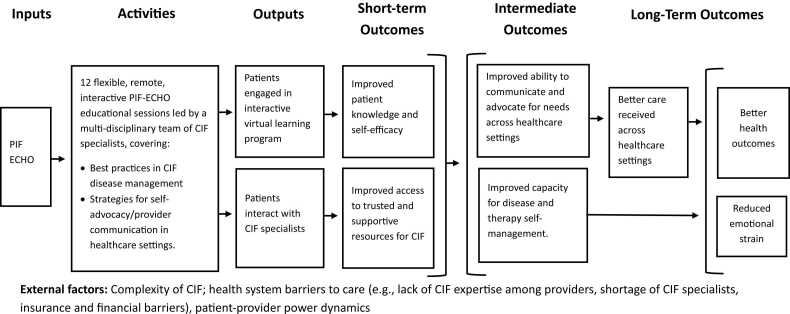
**Data-derived PIF-ECHO logic model illustrating pathways between proposed program activities and anticipated outcomes. External factors:** Complexity of CIF; health system barriers to care (e.g., lack of CIF expertise among providers, shortage of CIF specialists, insurance and financial barriers), patient-provider power dynamics. 1 *Methods Note: Logic model developed collaboratively by program and evaluation teams and incorporates interview and focus group findings.*

Findings from this study were incorporated into a logic model that illustrates the hypothesized pathways through which the PIF-ECHO program is expected to influence outcomes for patients with CIF, based on themes identified in this study (Fig. 1). The model depicts the connections between weekly PIF-ECHO sessions designed to address participant-identified needs (access to provider support, patient education related to disease self-management and self-advocacy) and anticipated program outcomes. Specifically, participation in the program is expected to increase knowledge, self-efficacy and access to CIF specialists, leading to improved patient capacity for disease self-management and self-advocacy in healthcare settings. These improvements are expected to then contribute to higher quality care, reduced emotional strain, and improved overall health.

## 5 Discussion

This formative qualitative study provides a patient-centered evidence base for the design of PIF-ECHO, demonstrating that patients with CIF have specific priorities that a structured virtual learning program is well-positioned to address. We found consistent and strong concerns about access to quality care and specialty expertise for CIF; burdens associated with navigating the healthcare system and self-advocacy; challenges in patient-provider communication and fear of being dismissed by medical providers; insufficient knowledge and a lack of patient information for self-care management; and desire for best practice and highly specialized information, education and resources to help patients manage their own care and treatment. Participants described multiple pathways through which the PIF-ECHO program could ameliorate some of these challenges and, ultimately, contribute to better health outcomes for patients with CIF, leading to the PIF-ECHO logic model.

In a complex rare disease like CIF, disparity in access to expert care is a matter of health equity [39]. Many patients must travel very long distances to see clinicians with the highly specialized knowledge required to treat their disease [39]. Advocates and focus group participants openly discussed the strains associated with not being able to find expert clinicians willing to treat them, as well as the emotional and financial costs and resources required to travel or relocate out of state for care at an IRP or another institution. Additionally, they described knowledge gaps in CIF and PN by medical specialists involved in their care and perceived their own situation as being too complex for hospitalists or primary care physicians. This viewpoint is consistent with a severe lack of knowledge in CIF and PN management found among gastroenterologists who self-identified as “experts” or “non-experts” [40]. Irrespective of exact cause, the lack of widely available expertise likely results in significantly impaired health outcomes for patients with CIF.

Advocates and focus group participants alike expressed a strong desire for more training and education to support their ability to advocate for and manage their own care. Confidence in CIF patients’ own ability to self-perform PN procedures is associated with understanding of the need for PN, how to care for the central line, knowing the signs and symptoms of central line infection, and knowing whom to call for therapy and disease-related questions [8]. Advocates and focus group participants also described searching for guidance and frequently turning to social media for peer advice to troubleshoot care of the central line. Use of social media by patients and caregivers to seek advice and support from peers has been reported elsewhere. For example, in a recent analysis of more than 300 social media posts by adult home PN patients, the most prevalent theme identified was “best practices, care, and safety of PN use;” and these posts primarily focused on central line care, choice of vascular access device, infection exposure, PN composition, and infusion schedules [41]. Similarly, the theme of “difficulty with PN expertise and access” was also prominent and included posts for locating physicians with PN expertise in specific geographic regions and access to specialty care [41].

Communication between patients and their providers emerged as an area that requires particular attention in the development of a PIF-ECHO program. Communication is essential in patient-centered care where the focus is on building solid partnerships between the patient, family, and clinician to combine medical expertise with the unique lived experience [36], [42]. Nevertheless, advocates and focus group participants alike described myriad challenges patients with CIF face in their communication with providers, especially those without specialized expertise. They noted that fear of being labeled as an adversarial patient and dropped from a provider’s care limits their willingness to share information with providers and consistently suggested that the PIF-ECHO program incorporate communication and advocacy strategies and resources.

Overall, participants in the interviews and focus groups were very interested in an online program providing best practice education about CIF and PN care. The overwhelming sentiment was that greater access to information would empower patients to take better care of themselves. These findings are consistent with a growing evidence base supporting patient-facing digital health interventions in rare disease populations, where information asymmetry and geographical isolation create significant barriers to self-management.

## 6 Limitations

Several limitations in this study should be acknowledged. First, recruitment methods, which relied on patients self-identifying as interested in in PIF-ECHO, likely introduced selection bias as volunteers may hold more favorable views of virtual education than the broader CIF population. Second, the focus group sample exclusively identified as female and white, and Medicaid participation—an indicator of socioeconomic status—was low. Lack of sample variation suggests need for caution in generalizing the findings to populations with greater gender, racial, ethnic, and socioeconomic diversity, who may face additional barriers to care and may have distinct programmatic priorities. Third, the small sample size reflects the pragmatic constraints of pre-intervention qualitative work in a rare disease population. Consequently, while findings are thought to be broadly representative of patients engaged with national CIF advocacy organizations and social media communities, they may not reflect the views of patients who are more isolated or less engaged with these networks. Additional studies, planned with larger and more diverse populations will address these limitations.

## 7 Conclusion

In summary, participants in this study reported barriers to accessing quality and expert care for CIF, a need for training in disease and therapy self-management, and a desire for more knowledge to empower confident self-advocacy and system navigation. Patients are very open and interested in multidisciplinary best practice education for chronic intestinal failure and shared recommendations for development and implementation of the PIF-ECHO program. These findings provide a patient-centered evidence base and framework for program development and establish the foundation for a formal evaluation of PIF-ECHO’s impact on clinical outcomes and quality of life in a randomized controlled trial.

## CRediT authorship contribution statement

**Rocco Friebel:** Writing – review & editing, Conceptualization. **Yiannoutsos Constantin T:** Writing – review & editing, Conceptualization. **Marjorie Nisenholtz:** Writing – review & editing, Resources, Project administration. **Ballog Princess B:** Writing – review & editing, Resources, Project administration, Data curation. **Malvika Nair:** Writing – review & editing, Writing – original draft, Methodology, Formal analysis, Data curation. **Marion Winkler:** Writing – review & editing, Writing – original draft, Formal analysis, Conceptualization. **Fisher Elisa M:** Writing – review & editing, Writing – original draft, Methodology, Formal analysis, Data curation, Conceptualization. **Iyer Kishore R:** Writing – review & editing, Writing – original draft, Resources, Project administration, Methodology, Funding acquisition, Conceptualization.

## Statement for studies in humans/animals

All interview and focus group participants provided verbal consent for taking part in the study and received modest incentives for their participation. Ethics approval has been obtained from the New York Academy of Medicine Institutional Review Board (IRB) with the letter number #011426 dated January 20, 2026, and from the BRANY IRB with letter number STUDY−24–01292 dated November 12, 2025.

## Funding

This work was supported by the 10.13039/100000133Agency for Healthcare Research and Quality (AHRQ) under award number 1R03HS030321−01.

## Declaration of Competing Interest

Marion Winkler reports a relationship with Ironwood Pharmaceuticals that includes consulting (Patient Leadership Council) or advisory. All other authors declare that they have no known competing financial interests or personal relationships that could have appeared to influence the work reported in this paper.

## References

[1] Dudrick S.J., Wilmore D.W., Vars H.M., Rhoads J.E. (1969). Can intravenous feeding as the sole means of nutrition support growth in the child and restore weight loss in an adult? An affirmative answer. Ann Surg.

[2] Wilmore D.W. (1969). Dudrick SJ. Safe long-term venous catheterization. Arch Surg.

[3] Dudrick S.J., Wilmore D.W., Vars H.M., Rhoads J.E. (1968). Long-term total parenteral nutrition with growth, development, and positive nitrogen balance. Surgery.

[4] Pironi L., Arends J., Baxter J., Bozzetti F., Peláez R.B., Cuerda C. (2015). Home Artificial Nutrition & Chronic Intestinal Failure; Acute Intestinal Failure Special Interest Groups of ESPEN. ESPEN endorsed recommendations. Definition and classification of intestinal failure in adults. Clin Nutr.

[5] Howard L., Heaphey L.L., Timchalk M. (1986). A review of the current national status of home parenteral and enteral nutrition from the provider and consumer perspective. JPEN J Parenter Enter Nutr.

[6] Howard L. (1989). Home nutritional support: the patient point of view. Nutr Clin Pract.

[7] Baxter J.P., Fayers P.M., Bozzetti F., Kelly D., Joly F., Wanten G. (2019). An international study of the quality of life of adult patients treated with home parenteral nutrition. Clin Nutr.

[8] Winkler M.F., Machan J.T., Xue Z., Compher C. (2020). Home Parenteral Nutrition Patient-Reported Outcome Questionnaire: Sensitive to Quality of Life Differences Among Chronic and Prolonged Acute Intestinal Failure Patients. JPEN J Parenter Enter Nutr.

[9] Winkler M.F., Smith C.E. (2015). The impact of long-term home parenteral nutrition on the patient and the family: achieving normalcy in life. J Infus Nurs.

[10] Kelly D.G., Tappenden K.A., Winkler M.F. (2014). Short bowel syndrome: highlights of patient management, quality of life, and survival. JPEN J Parenter Enter Nutr.

[11] Santarpia L., Buonomo A., Pagano M.C., Alfonsi L., Foggia M., Mottola M. (2016). Central venous catheter related bloodstream infections in adult patients on home parenteral nutrition: Prevalence, predictive factors, therapeutic outcome. Clin Nutr.

[12] Pironi L., Corcos O., Forbes A., Holst M., Joly F., Jonkers C. (2018). ESPEN Acute and Chronic Intestinal Failure Special Interest Groups. Intestinal failure in adults: Recommendations from the ESPEN expert groups. Clin Nutr.

[13] Allan P., Stevens P., Chadwick P., Teubner A., Abraham A., Carlson G. (2016). Osteomyelitis in adult patients on long-term parenteral nutrition: 2745 patient-years of experience in a national referral centre. Clin Nutr.

[14] Huard G., Fiel M.I., Moon J., Iyer K., Schiano T.D. (2018). Prevalence, Evolution, and Risk Factors for Advanced Liver Fibrosis in Adults Undergoing Intestinal Transplantation. JPEN J Parenter Enter Nutr.

[15] Buchman A.L., Iyer K., Fryer J. (2006). Parenteral nutrition-associated liver disease and the role for isolated intestine and intestine/liver transplantation. Hepatology.

[16] Horslen S.P., Sudan D.L., Iyer K.R., Kaufman S.S., Iverson A.K., Fox I.J. (2002). Isolated liver transplantation in infants with end-stage liver disease associated with short bowel syndrome. Ann Surg.

[17] Sasdelli A.S., Agostini F., Pazzeschi C., Guidetti M., Lal S., Pironi L. (2019). Assessment of Intestinal Failure Associated Liver Disease according to different diagnostic criteria. Clin Nutr.

[18] Pironi L., Sasdelli A.S. (2019). Intestinal Failure-Associated Liver Disease. Clin Liver Dis.

[19] Siddiqui M.T., Al-Yaman W., Singh A., Kirby D.F. (2020). Short-Bowel Syndrome: Epidemiology, hospitalization trends, in-hospital mortality, and healthcare utilization. JPEN J Parenter Enter Nutr.

[20] Iyer K.R., Kunecki M., Boullata J.I., Fujioka K., Joly F., Gabe S. (2017). Independence from parenteral nutrition and intravenous fluid support during treatment with teduglutide among patients with intestinal failure associated with short bowel syndrome. JPEN J Parenter Enter Nutr.

[21] Matarese L.E., Jeppesen P.B., O'Keefe S.J. (2014). Short bowel syndrome in adults: the need for an interdisciplinary approach and coordinated care. JPEN J Parenter Enter Nutr.

[22] DiBaise J.K., Young R.J., Vanderhoof J.A. (2004). Intestinal rehabilitation and the short bowel syndrome: part 2. Am J Gastroenterol.

[23] Iyer K., DiBaise J.K., Rubio-Tapia A. (2022). AGA Clinical Practice Update on Management of Short Bowel Syndrome: Expert Review. Clin Gastroenterol Hepatol.

[24] Neumann M.L., Allen J.Y., Kakani S., Ladner A., Rauen M.H., Weaver M.S. (2022). A beautiful struggle: Parent-perceived impact of short bowel syndrome on child and family wellbeing. J Pediatr Surg.

[25] Arora S., Thornton K., Murata G., Deming P., Kalishman S., Dion D. (2011). Outcomes of treatment for hepatitis C virus infection by primary care providers. N Engl J Med.

[26] Arora S., Geppert C.M., Kalishman S., Dion D., Pullara F., Bjeletich B. (2007). Academic health center management of chronic diseases through knowledge networks: Project ECHO. Acad Med.

[27] Arora S., Kalishman S., Thornton K., Dion D., Murata G., Deming P. (2010). Expanding access to hepatitis C virus treatment--extension for community healthcare outcomes (ECHO) project: disruptive innovation in specialty care. Hepatology.

[28] Jayasekera C.R., Arora S., Ahmed A. (2016). Hepatitis C treatment delivery mandates optimizing available health care human resources: A Case for Task Shifting. JAMA.

[29] Arora S. (2019). Project ECHO: democratizing knowledge for the elimination of viral hepatitis. Lancet Gastroenterol Hepatol.

[30] Salgia R.J., Mullan P.B., McCurdy H., Sales A., Moseley R.H., Su G.L. (2014). The educational impact of the Specialty Care Access Network-Extension of Community Healthcare Outcomes program. Telemed J E Health.

[31] Komaromy M., Bartlett J., Zurawski A., Gonzales-van Horn S.R., Kalishman S.G., Ceballos V. (2020). ECHO care: providing multidisciplinary specialty expertise to support the care of complex patients. J Gen Intern Med.

[32] Arora S., Kalishman S., Thornton K., Komaromy M., Katzman J., Struminger B. (2016). Project ECHO (Project Extension for Community Healthcare Outcomes): A National and Global Model for Continuing Professional Development. J Contin Educ Health Prof.

[33] Struminger B., Arora S., Zalud-Cerrato S., Lowrance D., Ellerbrock T. (2017). Building virtual communities of practice for health. Lancet.

[34] Arora S., Kalishman S.G., Thornton K.A., Komaromy M.S., Katzman J.G., Struminger B.B. (2017). Bradford AM. Project ECHO: A Telementoring Network Model for Continuing Professional Development. J Contin Educ Health Prof.

[35] Iyer K., Nisenholtz M., Gutierrez D., Winkler M., Tappenden K., Seidner D. (2021). Disseminating Knowledge in Intestinal Failure – Initial Report of the Learn Intestinal Failure Tele-ECHO (LIFT-ECHO) Project. JPEN J Parenter Enter Nutr.

[36] Kumpf V.J., Neumann M.L., Kakani S.R. (2023). Advocating for a patient- and family centered care approach to management of short bowel syndrome. Nutr Clin Pract.

[37] World Health Organization. Framework on integrated, people-centred health services: report by the Secretariat. (A69/39). 2016. Available from: https://apps.who.int/gb/ebwha/pdf_files/WHA69/A69_39-en.pdf. Accessed 2026.

[38] Colorafi K.J., Evans B. (2016). Qualitative Descriptive Methods in Health Science Research. HERD.

[39] Mundi M.S., Mercer D.F., Iyer K., Pfeffer D., Zimmermann L.B., Berner-Hansen M. (2022). Characteristics of chronic intestinal failure in the USA based on analysis of claims data. JPEN J Parenter Enter Nutr.

[40] Iyer K.R., Winkler M., Zubizarreta N., Nisenholtz M., Lucero K., Lubarda J. (2021). Knowledge of chronic intestinal failure among US gastroenterologists: Cause for concern and learning opportunity. JPEN J Parenter Enter Nutr.

[41] Barrera R., Poindexter K., Tucker C., Winkler M.F., Dashti H.S. (2024). Amplifying the lived experiences of parenteral nutrition consumers through the thematic analysis of social media posts. Nutr Clin Pract.

[42] Santana M.J., Manalili K., Jolley R.J., Zelinsky S., Quan H., Lu M. (2018). How to practice person-centred care: a conceptual framework. Health Expect.

