# Supplementary material for: Patient perspectives on barriers to care and the perceived value of a direct-to-patient virtual learning program for chronic intestinal failure
Source: Intest Fail. 2026 Aug 7;11:100390. doi: 10.1016/j.intf.2026.100390 (PMC13476477; doi:10.1016/j.intf.2026.100390)
Supplement: Supplementary file 1 [file mmc1.docx]

| **Supplementary Table 1. Topics of interest for PIF-ECHO sessions** | | |
| --- | --- | --- |
| **Topic** | **Sub-topic** | **Representative Quotes** |
| Resources, tools, and skill building | Identification of informational and clinical resources | *Databases of doctors who specialize in intestinal failure, or other aspects, like surgery. There are surgeons that will take cases that are really difficult and risky, whereas others won’t. (Focus group participant)*  *Navigating the system or accessing doctors that are not local. (Advocate)*  *I would try to teach people how to search quality information and how to find good people who can fact check with you. (Advocate)* |
| Resources, tools, and skill building | Self-advocacy and provider communication | *How do you actually get doctors to listen to you and how do you get them to actually believe what you’re saying? You can sound educated talking about it, and they still might not be receptive to the information. How to navigate that situation to make them more receptive to whatever information is learned through the program. (Focus group participant)*  *I think that if patients…can learn to articulate a quality-of-life goal and specifically ask for things that can improve their quality of life, that then they can have meaningful conversations. So, that might be an objective that you could have with this ECHO. (Advocate)*  *Really helping that person feel empowered to do the advocacy is actually gonna be more beneficial than like a checklist.* *I think, patients wanna feel that they’re validated in their desires. And that it’s okay to advocate and talk through some ways to do that for some specific goals that they have. It’s a learned skillset. (Advocate)* |
| Resources, tools, and skill building | Systems navigation supports and resources | *There are patient advocates, and for some of the expensive drugs, there are programs…that will help you with co-pays and, in some cases, premiums. [Learning about] that kind of stuff would also be very useful. (Focus group participant)*  *Access to those resources for help in navigating care, too, with rebates and things like that is also really helpful. I’ve had trouble trying to find some of that information. (Focus group participant)* |
| Access to disease-related information | — | *I think, first of all, just understanding that there’s the basis of diagnosis, basic understanding of the causes of that. And then, the management options that may be most appropriate. That would kinda be the way that I’d like look at the layers. And then, you can build from there. (Advocate)*  *That should probably be in the first [session], like basics, what intestinal failure is; why we're doing this; the end goal is to make things better, and try to improve people's chances on TPN, so they're not getting constant infections. (Advocate)*  *So, I think if there is better peer-reviewed information out there, readily access to that would be really nice. Unless you have access to these medical journals and things like that and know how to read them and decipher them, you’re not going to have the same access to the information. (Focus group participant)* |
| Treatment options | — | *While doing webinars or focus groups, we learn that there are new drugs in development…I would love that kind of information...Even if it’s some years down the road, I look forward to knowing that something’s coming down the pike. (Focus group participant)*  *And so, I don’t know what specific medications are, but even just [learning about] some of the newer things that are out there [and the] pros and cons, because it’s hard to know where to get good information of safety in terms of balance with effectiveness. (Focus group participant)* |
| Disease management | Central line care and management | *I think that basics of line care, like recognizing complications, is something important…I think you need to teach it with a trauma-informed perspective. “We know that you don’t want to go back to the hospital, but this is why [you may need to]. Sepsis really is dangerous.” They may not have gotten good training on infection prevention protocols. (Advocate)*  *[Patients] knowing what to look out for, whether they have a home health nurse coming in; they need to make sure to ask [for training]…They can ask their doctor to put in an order, so that the nurse teaches them how to do dressing changes, and things like that. (Advocate)*  *Line care, [including] when to go to the emergency room (Advocate)*  *Vascular access issues (Focus group participant)* |
| Disease management | Diet and nutrition | *Diet has been another issue where I had a really hard time navigating because there’s no clear answers. It is very unique to each individual, and we’re all going to be affected. (Focus group participant)*  *From a more clinical side, nutrition and how much it matters and output – nutrition intake and output, but how much that impacts your entire body but then your gut. So many people don't understand that your output is literally the cause of your kidney decline…but also your nutrition status. (Advocate)* |
| Quality of life | Living with CIF | *Any discussion that you have about safety should incorporate quality of life… You can’t teach someone to always prepare their TPN in one consistent place in their house and not also consider that they may want to travel. You have to teach them how to safely travel if you want them to truly be safe. (Advocate)*  *The parents want to know more about how active their kids can be. Or if they are active and playing sports, what are those parents [can do] to protect their kid’s line or make sure that they’re safe…we’ve still gotta bathe, we still gotta shower and stuff like that. And so, you kinda have to have workarounds, and some of them work and some of them don’t, I noticed. So, that’d be a topic. (Advocate)* |
| Quality of life | Navigating Relationships | *Even in support groups, we’re not talking about intimacy, relationships, how to navigate being together with lines, and having diarrhea, and these issues. How do you still have that intimacy within your marriage? Within your relationships? (Focus group participant)*  *I think [social] activity would be a good topic, because I find it very difficult…everything’s gonna revolve around food. How can we kinda change that perspective a little bit so that we can be more participatory in those holidays and those friendships and those relationships? (Focus group participant)* |
| Mental health support | — | *We’re all in fight or flight mode and that affects so much of it when you’re just not emotionally regulated…[there] are a couple psycho-social things [that] are so crucial to just surviving another day or having that capacity. And [they] can get professionals that we don't always interact with but they can come and speak. (Advocate)*  *Talking about combating burnout, because we don’t get to take days off. We don’t get to take hours off. You don’t have a choice…it can be really difficult when so many hours of your day, of your already very limited energy, is directed to just doing your daily care. (Focus group participant)* |
| *CIF: Chronic intestinal failure*  *ECHO: Project ECHO^®^ (Extension for Community Healthcare Outcomes)*  *PIF-ECHO: The Patient Intestinal Failure-ECHO Project*  *TPN: Total parenteral nutrition* | | |
